# Comparison of Intra-articular Steroid and Platelet-Rich Plasma Injections in Patients With Knee Osteoarthritis

**DOI:** 10.7759/cureus.48370

**Published:** 2023-11-06

**Authors:** Cigdem Cinar, Nazire Bagatir

**Affiliations:** 1 Department of Physical Therapy and Rehabilitation, Biruni University, Istanbul, TUR; 2 Department of Physical Therapy and Rehabilitation, Istanbul Physical Therapy Rehabilitation Training and Research Hospital, Istanbul, TUR

**Keywords:** quality of life (qol), steroid, platelet-rich plasma, osteoarthritis, knee

## Abstract

Introduction: This study aimed to compare the effects of intra-articular steroid (IAS) and platelet-rich plasma (PRP) injections on femoral cartilage thickness and quality of life in patients with knee osteoarthritis (OA).

Methods: This research was designed as a retrospective study with propensity score matching and included 80 treatment-naive patients with knee OA. All patients’ demographic characteristics and Visual Analog Scale (VAS), Western Ontario and McMaster Universities Osteoarthritis Index (WOMAC), and Short Form-36 (SF-36) scores before treatment, one month after treatment, and six months after treatment were recorded. Femoral cartilage thickness measurements were taken with ultrasonography. To determine the efficiency of IAS and PRP injections, data was collected before treatment, one month after treatment, and six months after treatment and then compared.

Results: All VAS values and WOMAC scores were significantly better one month and six months after treatment in both the IAS group and PRP group. However, IAS treatment was associated with significantly lower VAS scores during the night and during movement one month after treatment (p=0.044 and p=0.042). VAS at rest and the WOMAC scores one month after treatment were similar (p=0.435 and p=0.616). All VAS scores (night, rest, and movement) and WOMAC scores were comparable between groups six months after treatment (p=0.569, p=0.504, p=0.584, and p=0.441). There were no significant differences in femoral thickness at the eight measurement points between the groups at the follow-ups.

Conclusion: Our findings have shown that IAS and PRP injection treatments have a significantly positive effect on the pain and quality of life of patients with knee OA, although this positive effect for pain was significantly better for IAS at night and during movement at the one-month follow-up. Additionally, IAS and PRP injections had positive and comparable healing impacts on femoral cartilage thickness.

## Introduction

Osteoarthritis (OA) is a chronic, degenerative, and progressive joint disease that results in cell damage, an increased inflammatory response, and extracellular impairment [1]. Although OA can be detected in any joint, the knee joint is one of the most common areas in which the condition is diagnosed. Due to various factors, such as an increase in obesity prevalence, a more sedentary lifestyle, and a prolonged lifespan, OA has been diagnosed more frequently in recent decades [2]. Previous reports have demonstrated that almost 60% of the population faces OA after 75 years of age [3]. Another study found that one out of every five individuals had complaints about OA and OA-related complications after 45 years of age [4]. At the same time, numerous studies have shown the relationships between OA and the restriction of physical movement, increased drug use, increased hospital admission, loss of labor, and social isolation [3-5]. Furthermore, OA occurring in one joint may lead to deterioration of the structure of another joint due to limited movement or pain.

At present, OA can be treated with physical therapy, intra-articular injections, and surgery. Intra-articular treatments have come to the fore in recent years for reasons such as minimal pain, ease of application, and no hospitalization being required. In one study, Raeissadat et al. applied intra-articular injections of platelet-rich plasma (PRP) in patients with knee OA and concluded that PRP improved OA-related symptoms for 12 months [6]. In another study, Maricar et al. analyzed the effect of intra-articular steroid (IAS) treatments in patients with OA, with results indicating that tenderness around the joint was significantly improved following IAS injection [7].

Although previous reports have evaluated the efficiency of IAS and PRP injections, especially in knee OA, no studies have analyzed the effects of IAS and PRP injections on femoral cartilage thickness and quality of life in patients with this condition. In this study, we compared the effects of IAS and PRP injections on femoral cartilage thickness and quality of life in patients with knee OA.

## Materials and methods

This research was designed as a retrospective study with propensity score matching and included 80 patients with primary knee OA who applied to the same academic center between September 2017 and September 2019. Patient data was accessed using the hospital database. Ethical approval for the study was provided by the local ethics committee (decision no: 2020-18-18; approval no: 2018/181). All patients were informed in detail about the effectiveness and possible side effects of IAS and PRP treatments, and written informed consent was obtained from all patients. Inclusion criteria for the study were being diagnosed with primary knee OA, being over 45 years of age, being diagnosed with OA for at least one year, and the presence of level 2 or level 3 OA according to Kellgren-Lawrence staging on radiographs taken in the last six months. Exclusion criteria consisted of being diagnosed with secondary OA, having a history of a knee operation, having received physical therapy in the last three months, having received an intra-articular injection into the knee joint in the last three months, and having experienced serious trauma in the last three months. Also, patients with a history of serious psychological trauma in the last three months were excluded from the study.

The participating patients were divided into the IAS group and the PRP group in a 1:1 ratio. All patients’ characteristics, including age, gender, educational status, occupation, body mass index (BMI), side of the affected knee, disease duration (months), and X-ray radiograph properties according to the Kellgren-Lawrence staging system were recorded. Patients’ quality of life and pain scores were analyzed via the Visual Analog Scale (VAS) and Western Ontario and McMaster Universities Osteoarthritis Index (WOMAC) scores before treatment, one month after treatment, and six months after treatment. Also, patients’ VAS results were evaluated at three different times, which included during the night, during rest, and during movement. Additionally, all patients filled out Short Form-36 (SF-36) before treatment, one month after treatment, and six months after treatment. Femoral cartilage thickness measurements were taken with ultrasonography, and eight points were measured for each patient (right medial, right intermedial, right lateral, right mean, left medial, left intermedial, left lateral, and left mean). These measurements were performed before treatment, one month after treatment, and six months after treatment.

The WOMAC questionnaire and SF-36

The WOMAC questionnaire, which includes 24 items, is used to define the seriousness of hip and knee OA. Each item is scored between zero and four (none, mild, moderate, severe, and extreme), and higher WOMAC scores are related to worse functional capability, stiffness, and pain [8]. The SF-36 is a self-reported form designed to evaluate a patient’s health condition, and it includes eight scaled scores (physical functioning, physical role functioning, emotional role functioning, vitality, mental health, social functioning, bodily pain, and general health). Overall, a high SF-36 score indicates a better state of health [9].

To determine the efficiency of IAS and PRP injections on femoral cartilage thickness and quality of life in patients with knee OA, patients’ demographic data, VAS results, WOMAC scores, SF-36 scores, and femoral cartilage thickness measurements were collected before treatment, one month after treatment, and six months after treatment and then compared.

Statistical analysis

Data were analyzed using the IBM SPSS version 22.0 (Armonk, NY: IBM Corp.). The normality distribution of the variables was examined using visual (histogram and probability plots) and analytical methods (Shapiro-Wilk test). Descriptive statistics were shown as means and standard deviations for normally distributed numerical data and as medians and minimum-maximum values (min-max) for non-normally distributed data. Nominal data were expressed as numbers and percentages. For comparisons between groups, the Mann-Whitney U test and the independent samples t-test were used. Results with a p-value below 0.05 were considered statistically significant.

## Results

A total of 80 patients were enrolled in the study, and each group included 40 patients. Patients’ characteristics, including age, sex, education level, BMI, OA grade according to the Kellgren-Lawrence classification, side of OA, and duration of OA, were similar between the groups (p=0.150, 0.431, 0.816, 0.794, 0.119, 0.294, and 0.371, respectively) (Table 1).

**Table 1:** Sociodemographic and clinical characteristics of patients. *Mean±standard deviation. **Median (minimum-maximum). IAS: intra-articular steroid; PRP: platelet-rich plasma

Variables		IAS (n=40)	PRP (n=40)	p-Value
Age (years)*		58.0±9.0	53.6±9.5	0.150
Sex, n (%)	Female	29 (72.5%)	33 (82.5%)	0.431
	Male	11 (27.5%)	7 (17.5%)	
Education level, n (%)	Illiterate	4 (10.0%)	4 (10.0%)	0.816
	Primary school	28 (70.0%)	26 (65.0%)	
	Secondary school	2 (5.0%)	4 (10.0%)	
	High school	6 (15.0%)	6 (15.0%)	
BMI (kg/m^2^)*		31.1±5.4	30.6±5.5	0.794
Kellgren-Lawrence, n (%)	Grade 2	16 (40.0%)	22 (55.0%)	0.119
	Grade 3	24 (60.0%)	18 (45.0%)	
Side, n (%)	Right	32 (80.0%)	26 (65.0%)	0.294
	Left	8 (20.0%)	12 (30.0%)	
	Bilateral	-	2 (5.0%)	
Duration of illness (months)**		12 (1-72)	12 (2-120)	0.371

All VAS values and WOMAC scores were significantly improved one month and six months after treatment in both the IAS group and PRP group. The VAS scores during the night, at rest, and during movement, as well as the WOMAC scores, were not significantly different between the IAS and PRP groups before treatment (p=0.211, 0.249, 0.926, and 0.644, respectively). However, IAS treatment was associated with significantly lower VAS scores during the night and during movement one month after treatment than PRP treatment (p=0.044 and 0.042). However, VAS at rest and the WOMAC scores one month after treatment were similar between the groups (p=0.435 and 0.616). In addition, all VAS scores (night, rest, and movement) and WOMAC scores were comparable between groups six months after treatment (p=0.569, 0.504, 0.584, and 0.441, respectively) (Table 2).

**Table 2:** Comparison of VAS and WOMAC values between IAS and PRP groups. *Mean±standard deviation. **Median (minimum-maximum). ***Significant difference between the pretreatment value and first month after treatment or sixth month after treatment value. IAS: intra-articular steroid; PRP: platelet-rich plasma; VAS: Visual Analog Scale; WOMAC: Western Ontario and McMaster Universities Osteoarthritis Index

Variables		IAS	PRP	p-Value
Before treatment	VAS-night*	5 (0-10)	6 (0-9)	0.211
	VAS-rest*	5 (0-8)	2 (0-8)	0.249
	VAS-movement*	4 (0-8)	4 (1-8)	0.926
	WOMAC**	50.6±19.7	53.2±15.3	0.644
First month after treatment	VAS-night*	1 (0-10)***	4 (0-8)***	0.044
	VAS-rest*	0 (0-6)***	0 (0-5)***	0.435
	VAS-movement*	1 (0-8)***	4 (1-8)***	0.042
	WOMAC**	34.4±23.5***	31.2±14.6***	0.616
Sixth month after treatment	VAS-night*	0 (0-8)***	0 (0-8)***	0.569
	VAS-rest*	0 (0-8)***	0 (0-8)***	0.504
	VAS-movement*	1 (0-8)***	3 (1-9)***	0.584
	WOMAC**	37.5±22.0***	32.5±19.0***	0.441

Also, improvements in SF-36 scores between the IAS and PRP groups one month and six months after treatment were similar. The distribution and analysis of the SF-36 scores in the IAS and PRP groups are summarized in Table 3.

**Table 3:** Distribution and analysis of SF-36 scores in IAS and PRP groups. *Mean±standard deviation. **Median (minimum-maximum). ***Significant difference between the pretreatment value and first month after treatment or sixth month after treatment value. IAS: intra-articular steroid; PRP: platelet-rich plasma; SF-36: Short Form-36

Variables		IAS	PRP	p-Value
Before treatment	Physical functioning*	44.4±24.7	45.0±18.7	0.940
	Role physical**	25 (0-100)	25 (0-100)	0.855
	Role emotional**	0 (0-100)	33 (0-100)	0.494
	Vitality*	35.5±21.2	36.1±18.8	0.917
	Mental health*	44.2±17.0	48.5±19.4	0.458
	Social functioning*	54.1±24.2	57.4±24.4	0.669
	Bodily pain*	33.0±26.1	29.8±17.3	0.649
	General health*	46.5±22.7	41.6±16.9	0.441
First month after treatment	Physical functioning*	66.0±23.8***	53.5±23.9	0.108
	Role physical**	75 (0-100)***	50 (0-100)	0.512
	Role emotional**	67 (0-100)***	100 (0-100)	0.988
	Vitality*	46.0±25.8	42.3±20.2	0.618
	Mental health*	55.7±17.3***	48.1±18.0	0.184
	Social functioning*	73.2±19.9***	62.6±23.3	0.135
	Bodily pain*	59.3±25.3***	51.2±21.4***	0.285
	General health*	52.1±25.2	45.2±14.4	0.293
Sixth month after treatment	Physical functioning*	57.8±25.6***	54.5±24.2	0.672
	Role physical**	75 (0-100)	25 (0-100)	0.331
	Role emotional**	67 (0-100)***	33 (0-100)	0.955
	Vitality*	40.5±27.0	46.4±21.3***	0.447
	Mental health*	48.6±21.1	51.7±17.3	0.610
	Social functioning*	65.8±23.0***	64.4±20.9	0.834
	Bodily pain*	52.0±27.4***	50.1±24.9***	0.828
	General health*	50.1±25.4	45.7±16.6	0.514

The femoral cartilage thicknesses at the eight points were similar between the IAS and PRP groups before treatment. Also, there were no significant differences in femoral thickness at the eight measurement points between the groups at the follow-ups. Femoral cartilage thickness measurements and their comparisons between the two groups are presented in Table 4.

**Table 4:** Femoral cartilage thickness measurements and analysis in IAS and PRP groups. *Mean±standard deviation. **Significant difference between pretreatment value and first month after treatment or sixth month after treatment value. IAS: intra-articular steroid; PRP: platelet-rich plasma

Variables		IAS	PRP	p-Value
Before treatment*	Right medial	2.2±0.5	2.2±0.7	0.957
	Right İntermedial	1.8±0.4	2.2±2.1	0.444
	Right lateral	1.9±0.5	1.6±0.5	0.079
	Right mean	2.0±0.3	2.0±0.8	0.880
	Left medial	2.4±0.6	2.1±0.6	0.185
	Left intermedial	1.8±0.6	1.9±0.6	0.371
	Left lateral	1.8±0.5	1.7±0.6	0.528
	Left mean	2.0±0.4	1.9±0.5	0.682
First month after treatment*	Right medial	2.4±0.6	2.4±0.5	0.821
	Right intermedial	2.0±0.6	2.0±0.2	0.757
	Right lateral	2.0±0.6	1.9±0.4	0.539
	Right mean	2.1±0.5	2.1±0.3	0.979
	Left medial	2.5±0.6	2.5±0.7**	0.934
	Left intermedial	2.2±0.5**	2.2±0.5	0.893
	Left lateral	2.1±0.5	2.0±0.3**	0.578
	Left mean	2.3±0.4**	2.2±0.4**	0.848
Sixth month after treatment*	Right medial	2.2±0.6	2.1±0.4	0.445
	Right intermedial	2.0±0.6	2.2±0.5	0.323
	Right lateral	2.0±0.6	1.8±0.3	0.143
	Right mean	2.1±0.5	2.0±0.3	0.672
	Left medial	2.4±0.7	2.1±0.3	0.172
	Left intermedial	2.2±0.5**	2.4±0.6**	0.192
	Left lateral	2.2±0.7**	1.9±0.3**	0.194
	Left mean	2.2±0.6**	2.2±0.3**	0.609

## Discussion

OA is a common health problem all around the world, and millions of people are admitted to professional healthcare providers due to OA and OA-associated complications [10]. It is generally accepted that the knee is the most common site of OA. Intra-articular OA treatments have increased in popularity due to their minimally invasive nature, less associated pain, and outpatient procedures [11]. However, we also know that deterioration of one joint could result in pathologies in another joint. Thus, we conducted a study that investigated the effects of IAS and PRP injections on femoral cartilage thickness and quality of life in patients with knee OA. Our findings revealed that both the IAS and PRP procedures significantly increased patients’ quality of life according to the VAS, WOMAC, and SF-36 scores, but the improvements of both groups were similar. In contrast, with IAS, we achieved significantly better VAS scores at night and during movement at the one-month follow-up. In addition, the healing of the femoral cartilage was comparable for the IAS and PRP groups.

Pain, stiffness of the joint, and movement restrictions are the main complaints of OA. Previous reports have demonstrated that both VAS and VOMAC scores are useful forms for evaluating the seriousness of OA [8,12]. Also, the SF-36 is widely used to assess the general health status of patients. However, studies investigating the effects of intra-articular treatments for OA have had conflicting results. For instance, Bennell et al. investigated the potential benefits of PRP for knee OA and found that PRP had no significant beneficial effect on OA over a placebo [13]. In contrast, Di Martino et al. conducted a study to determine the impact of intra-articular PRP on knee OA, concluding that the procedure significantly improved the functional condition of the knee and significantly decreased OA-related symptoms [14]. In another study, McCabe et al. emphasized that while IAS had beneficial effects on pain in patients with OA, the evidence in this respect was relatively insufficient [15]. In the present study, we found that both IAS and PRP injections significantly increased the patients’ quality of life and significantly decreased pain. However, IAS and PRP injections had no advantage over each other in terms of improving symptoms, except for IAS providing significantly better VAS scores at night and during movement at the one-month follow-up.

Restriction of movement or pain in one joint may cause intentional or unintentional overload on another joint, which may lead to deterioration in the second joint. Steultjens et al. investigated the factors affecting functional deterioration of the hip joint and found that the presence of joints with instability was a risk factor [16]. Moreover, Peat et al. analyzed factors related to changes in disability and pain for knee and hip joints and concluded that OA of one joint resulted in greater deterioration for another joint [17]. Pelletier et al. showed the positive effect of IAS injection on femoral cartilage thickness [18]. In a different study, Baki et al. reported that femoral cartilage thickness increased at a statistically significant level after PRP injection [19]. In the present study, IAS and PRP injections for knee OA improved femoral cartilage thickness; however, the femoral cartilage thickening effects were not statistically significant between groups. IAS injection contributes to symptom management thanks to its strong anti-inflammatory effect. On the other hand, PRP injection has a disease-curing effect by promoting cartilage repair and regeneration through the release of growth factors. Therefore, we think that both treatments have a positive effect on patients' quality of life.

This study has some limitations. First, the relatively small number of patients can be considered a limitation. Additionally, the study focused only on the short-term effects of IAS and PRP injections. The long-term effects of these procedures on femoral cartilage thickness in patients with knee OA may be the subject of a different study. Furthermore, a cost analysis was not performed here and thus could be conducted in a different study.

## Conclusions

Our findings have shown that IAS and PRP injection treatments have a significantly positive effect on the pain and quality of life of patients with knee OA. Although this positive effect for pain was significantly better for IAS at night and during movement at the one-month follow-up. Moreover, both IAS and PRP injections had positive and comparable healing impacts on femoral cartilage thickness. Present study findings should be provided by further prospective randomized studies.
